# Doxing Victimization and Emotional Problems among Secondary School Students in Hong Kong

**DOI:** 10.3390/ijerph15122665

**Published:** 2018-11-27

**Authors:** Qiqi Chen, Ko Ling Chan, Anne Shann Yue Cheung

**Affiliations:** 1 Department of Applied Social Sciences, The Hong Kong Polytechnic University, Hong Kong, China; qchen2333@gmail.com; 2 Faculty of Law, The University of Hong Kong, Hong Kong, China; anne.cheung@hku.hk

**Keywords:** doxing, victimization, depression, anxiety, stress, privacy

## Abstract

Doxing is the searching for and intentional disclosure of private information about a particular individual on the Internet without his or her consent, and is often used to exact punishment. The aim of this study was to investigate the associations between doxing victimization and emotional problems in secondary school students, paying particular regard to the impacts of different types of doxed information, the relationship between the perpetrators and victims of doxing, and the nature of the online platforms where doxing occurs. A sample of 2120 Hong Kong secondary school students of differing socioeconomic backgrounds participated in the study. The results show that almost all types of disclosed personal information result in negative feelings in victims, including depression, anxiety, and stress. Girls were also found to be more likely than boys to be doxed. Significant associations were found between emotional problems and the disclosure of mobile phone numbers and personal photos and videos; doxing conducted by schoolmates and anxiety and depression, and doxing through Instant Messenger and anxiety. Further exploration of integrated cyber violence prevention programs and research on the details of doxing are recommended.

## 1. Introduction

The increasing use of social networking sites such as Facebook, Twitter, and Instagram has significantly transformed our social interactions. Such sites have become an integral part of many young people’s daily social lives [1], and yet awareness of the need to protect data privacy does not seem to have kept pace with the sites’ development [2]. The disclosure of personal data, broadcasting by adolescents of their own lives, and ability to leave comments on others’ posts have not only greatly enabled the communication and dissemination of personal information but also severely undermined privacy [3]. Individuals are at greater risk of violence, both online and off, when their personal information is easily accessible [4].

Of the various types of problematic online behavior, doxing, which refers to “searching for and publishing private or identifying information about a particular individual on the Internet, typically with malicious intent,” is particularly worrying [5]. Doxed information may include such private or even intimate data as an individual’s name, residential address, and academic or business record, and personal photographs and videos [5]. Connections are often drawn and developed among disparate pieces of information gathered from several sources, thereby enabling inferences to be made about the victim’s identity and even physical location [6]. A recent systematic review revealed that approximately 25% of adolescents in the United States have reported experiencing online harassment and victimization, with 35% of harassment victims having received threatening posts and messages at some point [7].

The typical motives for doxing include holding the targeted individuals to account for their wrongdoings, humiliating them for some reason, and online stalking [5]. The need to seek social approval and status often intensifies information disclosure and other risk-taking behavior, and the victims of real-world violence may also use Internet-based tools to seek revenge [8]. In contrast to harassment in the physical world, doxing is generally done anonymously in cyberspace and can happen to anyone. When doxing victims are identified by their name and address, it becomes easier for other netizens to infer further identifiable information about them, which constitutes a breach of victims’ right to data privacy [5]. Furthermore, the information disclosed is often shared and easily accessed through blogs, social networking sites, email, and/or online forums, thereby facilitating collective cyberbullying [9].

The current literature examining the demographic characteristics of doxing perpetrators and victims is limited. With respect to gender variations, studies report that girls are more likely than boys to be involved in relational aggression both in the physical world and in cyberspace as perpetrators and victims, including, for example, spreading rumors and releasing private information about others, excluding targeted individuals from one’s peer group, and encouraging group members to ostracize victims through threats [4,10]. One study suggests that gender differences may depend on the channel and form of perpetration; for example, girls are more frequently targeted via email, whereas boys are more often bullied through text messaging [3]. Researchers have also revealed that prevalence rates of cyberbullying peak during middle school, as adolescents manage to establish their place in the social hierarchy and gain control over peers [11]. Doxing can have devastating consequences for its victims, who fear being harassed physically in the real world and emotionally on the Internet, and abusive doxing can last for years [8].

Social media users often feel uncertain about and uncomfortable with what they share owing to the unpredictable nature of online audiences, and are therefore cautious about the content and posts they share with others [12]. Consequently, when trusted others disclose personal information without consent, the result may be social-related anxiety and exaggerated fear, with the user-victims becoming worried about being the focus of unwanted attention and the target of unwelcome evaluation [13]. However, both the victims and perpetrators of cyberbullying are found to be at greater risk of depressive symptoms, anger, low self-esteem, self-harm, and suicidal ideation [14], as well as academic difficulties and problems with peer relations [15,16]. Social networkers in Eastern cultures may also experience stress over the potential loss of face arising from personal privacy leakages [13]. Doxing-assisted harassment may increase the risk of victims being physically harmed or cyberbullied when personal information is used to locate and abuse them [5].

The psychological effects of doxing seem to cause as much as or more emotional harm than real-world bullying owing to the users’ reliance on networking interactions, wider audience, and potentially devastating impacts of harassment and stalking in cyberspace [17]. However, no study to date has examined the impact of specific types of doxed information or specific types of doxing perpetrators on the specific form of emotional problems suffered by doxing victims. Our hypothesis in the study reported herein was that doxing is positively associated with psychological problems in adolescents and that the negative effects on adolescents vary with the type, form, and/or perpetrator of the doxing behavior. This analysis is the first in this area of research, and its aim is to further scholarly understanding of the impacts of doxing on adolescents’ mental health.

## 2. Methods

### 2.1. Study Design and Sampling

The study employed data from a school survey covering different types of schools and students of differing socioeconomic backgrounds in Hong Kong. One class from each Secondary 2 to Secondary 5 grade, equivalent to grades 8 to 11 in the US, was randomly sampled in each school, and all students in the four sampled classes were invited to participate in the survey. A total of 2120 students participated in the survey. The participants signed informed consent forms and completed questionnaires with self-report questions on their lifetime doxing victimization experience and past-week psychological feelings regarding victimization at school under the instruction of trained interviewers. There were slightly more male participants (*n* = 1123, 52.97%) than female (*n* = 997, 47.03%), and the average age was 15.11 (SD = 1.45). The Institutional Review Board of the University of Hong Kong granted ethical approval for the survey (Ethical Code: EA1602044).

### 2.2. Measures

#### 2.2.1. Demographic Characteristics

Participants’ demographic and socioeconomic characteristics were collected using a self-constructed questionnaire including items on their age, gender, education level, and the education level of their father and mother.

#### 2.2.2. Experiences of Doxing Victimization

The participants’ experiences of having their personal information disclosed by others without their consent were assessed by three self-constructed items on the type of information disclosed, the person who had made the disclosure, and the platform on which the disclosure had been made. All three items were multiple-selection items requiring participants to check all responses that applied in their case. The types of personal information disclosed without consent were also assessed by the number of times such disclosures had taken place, with the possible responses including never, 1–2, 3–6, 7–10, 11–15, and over 15 times. For descriptive analysis of the prevalence, the results were recoded as 1 (yes) if participants selected any number of times that such doxing had occurred and as 0 if they chose “never.”

#### 2.2.3. Depression, Anxiety, and Stress

The 21-item Depression Anxiety Stress Scale (DASS-21) is the short form of the 42-item self-report DASS [18]. It was employed in this study to assess depression, anxiety, and stress in the participating doxing victims. The participants were asked about their feelings if they had learnt that their personal information had been disclosed without their consent. The items were divided into three dimensions, and rated as (Never), 1 (Sometimes), 2 (Often), and 3 (Always). Total scores were determined by summing the scores for the relevant severity dimensions, including 1(Normal), 2 (Mild), 3 (Moderate), 4 (Severe) and 5 (Extremely Severe), with a specific cut-off score set for each dimension. The DASS-21 achieved good reliability, with a total Cronbach’s alpha of 0.93, and Cronbach’s alphas of 0.88, 0.82, and 0.90 for the Depression, Anxiety, and Stress scales, respectively.

### 2.3. Statistical Anzalysis

Participants’ demographic characteristics and the prevalence rates of doxing victimization were computed using descriptive statistics and divided by gender. Spearman’s coefficient values were presented to indicate the degree of associations between doxing victimization and the DASS total and subscale scores. *p* < 0.05 was considered statistically significant, and SPSS version 25.0 (SPSS Inc., Chicago, IL, USA) was used to perform all of the statistical analyses in this study.

## 3. Results

### 3.1. Types of Personal Information Disclosed and Associations with DASS Scores

Table 1 shows the prevalence of the various types of personal information being doxed without consent and comparison of each type by gender. Overall, 15–31% of participants reported that their personal information or data has been disclosed by others without their consent, including their personal photos or videos (31.4%), name (29.9%), birthday (24.2%), and mobile phone number (15.1%). Girls were more likely than boys to report doxing victimization with respect to all types of information except their Hong Kong ID number (all *p* < 0.05). Almost all personal information doxed without consent lead to victims’ emotional problems, among which the most significant associations were between mobile phone number and emotional problems (Spearman’s *r*s ranging from 0.125 to 0.148, *p* < 0.001), private conversation with stress (*r* = 0.129, *p* < 0.001), personal and embarrassing photos and videos and depression (rs ranging from 0.123 to 0.124, all *p* < 0.001).

### 3.2. Doxing Perpetrators and Associations with DAS

As shown in Table 2, over half the participants named classmates as the individuals who had perpetrated the doxing in question (50.7%, *p* < 0.05). All five perpetrator groups were familiars of the victims, including fellow students in the same grade (30.3%) or school (28.3%), other people they knew (26.2%), friends outside school (25.7%), and parents/family members (24.6%). With respect to victims’ DAS, the most significant associations were between doxing perpetrated by students in the same school and anxiety (*r* = 0.109, *p* < 0.001) and depression (*r* = 0.091, *p* < 0.01), and doxing by other people personally known to victims was also significantly associated with depression (*r* = 0.086, *p* < 0.01).

### 3.3. Doxing Platforms and Associations with DAS

The platforms on which participants had been doxed are reported in Table 3. Over half of all personal information had been released through Instant Messenger (61.3%) and social networking sites (54.9%), with girls reporting much higher rates of disclosure on these two platforms than boys (all *p* < 0.001). Boys were more likely to be doxed on the other platforms considered: email (4.9%), online forums (0.4%), and blogs (0.4%). The most significant associations with respect to DAS were those between being doxed via Instant Messenger and anxiety (*r* = 0.087, *p* < 0.01) and depression (*r* = 0.083, *p* < 0.01). Doxing victimization on social networking sites was also associated more with victims’ emotional problems (rs ranging from 0.63 to 0.68, all *p* < 0.05).

## 4. Discussion

Our study is the first to estimate the associations between doxing victimization and depression, anxiety, and stress suffered by adolescent victims. Employing a large school sample, the study contributes to our understanding of the impacts on victims’ emotional status of different types of doxing victimization, different types of doxing perpetrators, and different doxing platforms.

The results suggest that girls are more likely than boys to be doxed across the major types of information and doxing perpetrators. With respect to doxing platforms, the girls in this study reported much higher rates of doxing via Instant Messenger and social networking sites than boys, which is consistent with the findings of previous studies [19] reporting girls to be more likely than boys to be involved in indirect aggression. This could be explained by previous findings that compared with talking in person, girls are more comfortable interacting with others on social networking sites or through text messages than boys do [20]. However, some studies report equal amounts of victimization in cyberspace for boys and girls [3].

Our findings also revealed the disclosure of almost all types of information subject to doxing to be significantly associated with such negative emotional states as depression, anxiety, and stress. These results are in line with those of previous findings that cyber-victimization can be the catalyst of impaired mental health, distress, and fear and can even increase the risk of suicidal ideation [21]. The disclosure of such personal information as one’s name, address, or mobile phone number makes one easily identifiable, thereby provoking anxieties over identity theft or harassment, and the sharing of location-based information can also expose people to the risk of being physically harmed [22].

In addition to the foregoing potential risks that cause doxing victims to fear for their own personal safety or the safety of those close to them, victims may also worry about the unpredictable but largely negative social evaluation and judgment that unauthorized information disclosure exposes them to. Private information or data, such as one’s personal and embarrassing photos or videos, relationship status, and private Internet or text conversations, which are sometimes surprising or even embarrassing, can also be used for “cyber-lynching” and personal revenge [23]. These situations are likely to induce depression and stress in those being doxed, as the uncomfortable judgment of the unknown masses very often leads to serious repercussions for doxing victims [13].

We found that over half the information doxed in our study had been disclosed by the victims’ classmates, and participants who reported being doxed by schoolmates reported the most significantly negative emotional feelings. Compared with strangers and Internet friends, students have more frequent interactions with their classmates, who therefore have more opportunities to obtain personal information about them. Self-efficacy and outcome expectations are closely related to an individual’s selection of activities, and therefore people with a higher degree of social anxiety tend to avoid social disclosure [24]. Doxing victims may worry about receiving undesirable evaluations from their schoolmates, whose gossip and/or negative judgments bring social pressure and can cause misunderstandings to spiral out of control. The experience of being victimized can cause emotional harm to students, and those with negative feelings about themselves are also more likely to be further targeted for victimization [25].

Significant associations were found in our study between unauthorized disclosure via Instant Messenger, social networking sites and feelings of depression, anxiety, and stress in victims. Social networking sites and online chatting have become increasingly important alternatives to face-to-face interactions, particularly for socially anxious individuals, who may struggle to maintain relationships and integrate into social groups [26,27]. Self-disclosure is linked to concerns over privacy and trust, and people generally share intimate information only if they are sure that the recipients are trustworthy [28]. Social media allow users to post information about others, and may thus disrupt people’s control over their own self-presentation and conflict with individual impression management. A fruitful direction for future studies would be to explore and make comparisons among the social, personality, and psychological impacts of doxing victimization.

A variety of educational strategies have been implemented by schools globally in recent years to raise young people’s awareness of online risks and reduce their exposure to the associated negative experiences, with the I-SAFE Internet Safety Curriculum in the U.S. [29] and Cyber Friendly Schools Project [30] in Australia being notable examples. Further exploration of integrated cyber violence prevention programs is therefore strongly recommended for schools with regard to preventing doxing victimization. The study reported herein constitutes an initial step toward comparing the negative psychological impacts of various forms of doxing victimization, but further explorations are required to unravel the details of the relationship between doxing and emotional problems and to obtain more up-to-date information on online doxing. The participants recruited for this study were secondary school students in one city in East Asia, and they may not be representative of students from other cities, countries, or cultures. It is recommended that future researchers recruit samples with a greater variety of demographic characteristics, including participants with different types of personality, socioeconomic status, and family background, to compare the relationship between psychological reactions and doxing victimization.

## 5. Conclusions

The present study contributes to our understanding of the negative emotional impacts of doxing victimization in adolescents by taking into account the impacts of various doxing information types, perpetrators, and platforms. Although the unauthorized disclosure of most types of personal information on the Internet causes psychological suffering in victims, the associations with symptoms of anxiety, depression, and stress are most significant when the doxing is perpetrated by schoolmates via webpage and social networking sites. Female students are more vulnerable to doxing victimization than their male counterparts, regardless of the type of information disclosed. Awareness of healthy online communication and privacy protection are of great importance to reducing such detrimental activities as harassment, cyberbullying, and even physical harm in the real world.

## Figures and Tables

**Table 1:** Associations between types of personal information doxed (disclosed without consent) and DASS scores of victims (*n* = 2120).

Types of Doxed Information	Prevalence				DASS Correlation		
	Male (%)	Female (%)	Total (%)	Chi-Square	Depression	Anxiety	Stress
Personal photos or videos	27.7	35.5	31.4	34.811 ***	0.124 ***	0.108 ***	0.118 ***
Name	24.4	36.0	29.9	52.886 ***	0.101 **	0.086 **	0.074 *
Birthday	18.8	30.0	24.2	44.818 ***	0.106 ***	0.096 **	0.100 **
Mobile phone number	12.4	18.1	15.1	28.667 ***	0.148 ***	0.146 ***	0.125 ***
School name	10.5	19.5	14.8	47.820 ***	0.109 ***	0.117 ***	0.107 ***
Academic performance	7.1	12.1	9.5	30.035 ***	0.089 **	0.084 **	0.082 **
Locations	7.7	11.0	9.3	13.655 *	0.090 **	0.097 **	0.099 **
Private internet or text conversation	5.2	13.4	9.1	55.864 ***	0.123 ***	0.119 ***	0.129 ***
Embarrassing photos or videos	6.5	11.4	8.8	18.399 **	0.107 ***	0.111 ***	0.124 ***
Personal email address	6.8	9.2	8.0	8.300	0.096 **	0.083 **	0.089 **
Relationship status	4.2	9.1	6.6	25.169 ***	0.121 ***	0.115 ***	0.113 ***
Odd habits	3.6	7.2	5.4	18.065	0.105 ***	0.090 **	0.096 **
Parents’ names	4.6	4.2	4.4	3.250	0.113 ***	0.085 **	0.117 ***
Intimate photos or videos	3.3	5.3	4.3	7.073	0.085 **	0.085 **	0.102 ***
Student card	4.2	4.1	4.2	2.829	0.108 ***	0.092 **	0.102 **
Home telephone number	3.9	4.0	3.9	3.354	0.085 **	0.068 *	0.081 **
Home address	4.3	2.5	3.5	7.523	0.097 **	0.080 **	0.106 ***
Sexual orientation	2.4	3.4	2.9	4.108	0.120 ***	0.113 ***	0.115 ***
Usernames and passwords of online accounts	2.2	2.7	2.5	7.187	0.076 *	0.080 **	0.085 **
Religious beliefs	2.6	1.7	2.2	6.122	0.041	0.041	0.050
Passport number	2.5	1.3	1.9	7.496	0.062 *	0.042	0.074 *
Racial or ethnic origin	1.6	1.3	1.4	4.962	0.030	0.027	0.041
Political opinions	1.6	0.8	1.2	4.540	0.038	0.043	0.055
Obscene or indecent photos or videos	0.7	1.4	1.0	8.122	0.078 **	0.069 *	0.100 **
Sexual life	1.1	0.9	1.0	2.732	0.098 **	0.091 **	0.099 **
ID card number	1.0	0.9	1.0	17.710 **	0.024	0.005	0.045
Medical records	1.1	0.4	0.7	4.737	0.077 **	0.052	0.081 **
Bank account numbers	0.6	0.6	0.6	3.470	0.083 **	0.067 *	0.082 **

Note: * *p* < 0.05, ** *p* < 0.01, *** *p* < 0.001.

**Table 2:** People who conducted the doxing and associations with DAS of victims (*n* = 2120).

People Who Conducted Doxing	Prevalence				DASS Correlation		
	Male (%)	Female (%)	Total (%)	Chi-Square	Depression	Anxiety	Stress
Parents/family members	20.8	28.0	24.6	7.84 **	0.038	0.033	−0.006
Classmates	46.5	54.3	50.7	6.26 *	0.045	0.058 *	0.015
Other students in the same grade	28.8	31.6	30.3	0.60	0.078 **	0.067 *	0.048
Other students in your school	26.5	29.9	28.3	1.55	0.091 **	0.109 ***	0.059 *
Teacher/Tutor	2.7	3.2	2.9	0.27	0.019	0.031	0.026
Friends outside your school	20.6	30.2	25.7	13.18 ***	0.028	0.037	0.038
People you personally know	21.7	30.0	26.2	10.04 **	0.086 **	0.074 *	0.055
Internet friends	3.8	6.2	5.1	3.18	0.029	0.009	0.034
Strangers	3.5	4.6	4.1	1.24	0.019	0.038	0.029

Note: * *p* < 0.05, ** *p* < 0.01, *** *p* < 0.001.

**Table 3:** Platforms of doxing and associations with DAS of victims (*n* = 2120).

Platforms of Doxing	Prevalence				DASS Correlation		
	Male (%)	Female (%)	Total (%)	Chi-Square	Depression	Anxiety	Stress
Instant Messenger	53.7	67.8	61.3	21.91 ***	0.083 **	0.087 **	0.068 *
Social networking site	44.7	63.7	54.9	36.94 ***	0.063 *	0.068 *	0.066 *
Chatroom	9.7	8.8	9.2	0.05	0.049	0.050	0.018
Email	6.2	3.6	4.9	3.33	0.031	0.047	0.051
Video-sharing website	2.9	1.7	2.2	0.18	0.030	0.059 *	0.062 *
Webpage	1.5	1.5	1.5	0.00	0.022	0.029	0.016
Forum	0.7	0.2	0.4	2.17	0.032	0.027	0.049
Blog	0.5	0.3	0.4	0.33	0.032	0.027	0.047

Note: * *p* < 0.05, ** *p* < 0.01, *** *p* < 0.001.
